# Diagnostic algorithm and case conundrums: patients presenting with proximal muscle weakness

**DOI:** 10.1186/1471-2474-14-S2-O4

**Published:** 2013-05-29

**Authors:** Tiziana Mongini

**Affiliations:** 1 Center for Neuromuscular Diseases, Department of Neuroscience, University of Turin, Turin, Italy

## 

In the last years it has been observed that enzyme replacement therapy is mostly effective when the treatment is started at an early stage of the disease, before advanced damage has occurred in muscle fibers; consequently, the timing of diagnosis has acquired an increasingly important role, both in children and in adults. We are now aware that some LOPD cases may show for years only a persistent mild hyperCKemia with no signs or symptoms. Often the finding of high serum CK levels in this group of asymptomatic patients is incidental (visit in the emergency room for chest pain, preoperative investigations, detection of increased serum transaminases) and their diagnosis may be very difficult. Different is the case of patients who are seen when they start to manifest weakness, usually with onset in the pelvic girdle muscles. They usually report fatigue, exertional dyspnea (eg, in climbing stairs), difficulties to get up from the ground or sitting: however, these are very common symptoms, shared by a large number of primary or secondary neuromuscular disorders. In the absence of a family history of Pompe disease or a biopsy with typical vacuolar changes, the diagnosis is easily missed, and this explains the broad diagnostic delay still now registered, with consequences on ERT efficacy. Neuromuscular diseases that may present with isolated proximal weakness include lower motor neuron diseases (eg, spinal muscular atrophy), disimmune motor neuropathies, neuromuscular junction disorders (pre-and post-synaptic), and a large number of genetically defined (eg, progressive muscular dystrophies due to altered sarcolemma proteins, congenital myopathies, channellopathies) or acquired myopathies (inflammatory myopathies, toxic myopathies). Differential diagnosis is often difficult and expensive, and can be completed only in specialized tertiary centers. A diagnostic algorithm aimed to facilitate the evaluation of patients with limb-girdle muscular weakness is presented, to target the use of specific tests that can be implemented even in non-specialized structures (eg, units of general neurology or rheumatology) and allow an early detection of Pompe disease. A series of clinical cases is presented, to stimulate a discussion and to prove the validity of the algorithm.

